# Improving diagnostics of rare genetic diseases with NGS approaches

**DOI:** 10.1007/s12687-020-00500-5

**Published:** 2021-01-15

**Authors:** Mateja Vinkšel, Karin Writzl, Aleš Maver, Borut Peterlin

**Affiliations:** Clinical Institute of Genomic Medicine, University medical Centre Ljubljana, Zaloška cesta 7, Ljubljana, Slovenia

**Keywords:** Next generation sequencing, Rare diseases, Healthcare system, Diagnostics

## Abstract

According to a rough estimate, one in fifteen people worldwide is affected by a rare disease. Rare diseases are therefore common in clinical practice; however, timely diagnosis of rare diseases is still challenging. Introduction of novel methods based on next-generation sequencing (NGS) technology offers a successful diagnosis of genetically heterogeneous disorders, even in case of unclear clinical diagnostic hypothesis. However, the application of novel technology differs among the centres and health systems significantly. Our goal is to discuss the impact of the implementation of NGS in the diagnosis of rare diseases and present advantages along with challenges of diagnostic approach. Systematic implementation of NGS in health systems can significantly improve the access of patients with rare diseases to diagnosis and reduce the dependence of national health systems for cross-border collaboration.

## Introduction

Rare diseases present an important public health burden since they affect 6–8% of the EU population. It is estimated that about 80% of rare diseases have a genetic origin and new genomic technologies revolutionized diagnostic approach (European Commission n.d.).

Even though rare diseases have affected human health throughout the human history, uniform recognition of this disease group, including terminology did not emerge until this millennium (Richter et al. 2015). Diagnosing rare diseases has been a significant challenge in the past. The first diagnostics of rare diseases began in the early twentieth century and was based on biochemical parameters (Garrod 1902). Cytogenetic methods were developed in the 1960s, and the molecular genetics and molecular cytogenetic methods emerged in the last decades of the twentieth century. In the last decade, methods based on genome sequencing have emerged, and modern medicine has thus been given a remarkable tool to address the diagnostics of rare diseases (Durmaz et al. 2015).

Despite the vast development of new technologies, there are still several challenges related to the timely and successful discovery of the aetiology of the rare disease. Although each of the rare genetic disease is rare, Online Mendelian Inheritance of Man database (OMIM n.d.) records 5520 different monogenic disorders, associated with 3832 genes (OMIM - Online Mendelian Inheritance in Man n.d.). Additionally, there are at least 67 recognized genetic syndromes associated with genomic mutations, like deletions, duplications and other genomic rearrangements of the genome less than 17 Mb (DECIPHER).

Apart from the rarity of the individual genetic disease, part of the complexity also comes from their genetic and clinical heterogeneity. Namely, the exact or similar phenotype may be associated with different genetic mechanism and may be due to locus or allele heterogeneity; while the first indicates mutations in different genes, the latter implies different alleles in the same gene (Deng and Pan 2018; Posey et al. 2019). Clinical heterogeneity is reflected by the fact that the same mutation or mutations in the same gene may lead to different disease phenotypes. Moreover, in several genetic diseases, the genetic predisposition is not always penetrant or may have variable phenotypic expression. Clinical signs of genetic diseases are frequently not pathognomonic, which further complicates the identification of rare genetic diseases (Schacherer 2016; Wright et al. 2019; Rahit and Tarailo-Graovac 2020).

Finally, the rarity of genetic diseases presents an additional challenge for the establishment of the diagnosis, due to unfamiliarity of health workers at the primary, secondary, and frequently even at the tertiary health institutions. The problem is even more evident in countries with a small population, where the number of patients with rare diseases is small, and consequently, there is a deficit of infrastructure, coordination, resources, and knowledge of rare genetic diseases.

Consequently, the diagnostics of rare genetic diseases used to be relatively inefficient (Di Resta et al. 2018). Namely, with Sanger sequencing which used to be the golden standard approach to the identification of human pathologic genetic variation, only single genes could be analysed. Furthermore, in case of genetic heterogeneity, “gene by gene” genetic testing approach was used, which was time consuming, expensive and therefore not available in several health systems (Jamuar and Tan 2015; Reuter et al. 2015; Payne et al. 2018). Furthermore, the correct diagnostic hypothesis was essential for successful genetic diagnosis.

Next generation sequencing technology revolutionized genetic testing. It has significantly contributed to gene identification for Mendelian disorders. Until 1986, only approximately 40 genes responsible for genetic diseases were identified. Positional cloning approach, which included mapping the gene in the genome, cloning the region of interest and sequencing the candidate genes led to the identification of more than 1000 genes, including the genes for cystic fibrosis and Huntington disease (Collins n.d.; Kremer et al. 1994; Lipner and Greenberg 2018). After the first successful application of NGS for gene identification in 2010 (Ng et al. 2010), the number of genes associated with human Mendelian disorders increased exponentially, and by the year 2017, 87% of gene discoveries resulted from the use of this specific method (Bamshad et al. 2019).

Apart from the identification of new genes for human disorders, the use of NGS significantly improved diagnostics of rare genetic disorders and has transformed healthcare systems. This manuscript aims to discuss benefits, challenges and limitations of NGS as well as the implication of systematic use of NGS in health systems.

## NGS as a diagnostic tool

Clinical signs that facilitate the recognition of the diseases have limitations in diagnostics of rare genetic diseases since genetic disorders usually display wide phenotypic and genetic heterogeneity. Therefore, clinically oriented approach for diagnosis of genetic diseases is often not successful, unless the phenotype is highly specific for a genetic disease. Nevertheless, confirming the diagnosis at the molecular level is essential in modern clinical practice for appropriate management of patient and patient’s family.

NGS transformed the field of genetic disease diagnostics with rapid, high-throughput and cost-effective approaches. NGS can simultaneously analyse from a few to hundreds of genes, whole exome and even whole genome, which proves to be a significant advancement towards deciphering the genetic heterogeneity of rare diseases and enables the investigations of genes, that extends beyond the clinical hypothesis (Jamuar and Tan 2015; Sawyer et al. 2016; Martínez et al. 2017; Bergant et al. 2018).

Benefits of NGS apart from ending the “diagnostic odyssey”, may lead to various clinically relevant outcomes; such as a change in disease management, enhanced surveillance, prognosis expectations, medication or dietary changes, new specialists referral, stopping of unnecessary or invasive investigations, prenatal or preimplantation genetic diagnosis, preconception carrier screening and predictive genetic testing of asymptomatic relatives (Sawyer et al. 2016; Stark et al. 2016; Tan et al. 2017; Malinowski et al. 2020).

Despite a significant leap in diagnostics of rare genetic diseases in recent years, more than half of patients with the suspected rare genetic disease remain without a definite diagnosis (Clark et al. 2018). There is an estimation that 200 million people worldwide are living with an unresolved genetic disease, but likely the number is even more significant due to the nonrecognition of genetic diseases (Global Genes n.d.).

Eurordis study found that 25% of patients with a rare disease are waiting from 5 to 30 years for confirmatory diagnosis and during this time 40% receive a misdiagnosis (EURORDIS—The Voice of Rare Disease Patients in Europe). Undiagnosed patients with genetic diseases are a particularly vulnerable group of patients with specific unmet needs. Patients with a rare disease who are not diagnosed or diagnosed late may have a delay for the start of a specific treatment, which, in turn, could have irreversible consequences for their health; may prevent the choice of informed reproductive choice and could cause great stress for patients and their families. Many European and international projects and initiatives have been created to support an undiagnosed patient with a genetic disease (EURORDIS - The voice of rare disease patients in Europe n.d.; Global Commission to End the Diagnostic Odyssey for Children with a Rare Disease n.d.; RD-Connect n.d.; Solve-RD n.d.; UDN | Undiagnosed Diseases Network n.d.).

## NGS approaches: brief overview

Three main sequencing approaches are used in clinical settings and indicated for the detection of rare variants in patients with a phenotype suspected to be due to a Mendelian disease; targeted sequencing panels, whole exome sequencing (WES) and whole genome sequencing (WGS).

### Targeted sequencing panel

Gene panels have a select number of specific genes or coding regions within genes that are known to harbour mutations that contribute to the pathogenesis of a certain disease or disease group. Targeted panels can be sequenced at a greater depth than WES and WGS and with lower cost and a significant reduction in turnaround times. Detected variants are limited to selected genes and produce a lower volume of data; consequently, the workload required for interpretation is lower, and there is a minimal concern of returning the incidental findings and variants of unknown significance in comparison to WES and WGS. However, due to new knowledge and gene discoveries, panels require regular updates. Panels and WES are limited in detection of structural variants, repetitive elements and mitochondrial genomic variants (Xue et al. 2015; Klein and Foroud 2017; Fernandez-Marmiesse et al. 2017; Lionel et al. 2018; Bean et al. 2020).

### Whole exome sequencing

Whole exome sequencing investigates protein-coding regions of the genome, which covers 1–2% of whole genome and harbours about 95% of disease-causing mutations (Posey 2019). WES enables the identification of variants in genes not yet associated with human genes. WES can be interpreted with a preselected panel or with a selected set of genes. The laboratory can select bioinformatics panel that includes a list of genes, which are known to be associated with the patients’ phenotype (Wang et al. 2019). The second approach is where laboratory examines all rare and potentially damaging variants and compares the phenotypes associated with these genes with the patient phenotype. The latter approach enables the identification of variants in genes not yet discovered (Ales et al. 2016; Reuter et al. 2017; Monies et al. 2019; Bruel et al. 2019). Limitations of the WES approach are due to the incomplete coverage of the regions, limited ability for detection of variation in repetitive elements, causal variants in cases of somatic mosaicism, structural variants and deep intronic variants (Xue et al. 2015; Seaby et al. 2016; Stark et al. 2016; Caspar et al. 2018; Lalonde et al. 2020). Nevertheless, with the advancement of technology, the exon coverage has improved, and the method also allows for improved coverage of regions outside the exons, including all known disease-causing intronic variants.

### Whole genome sequencing

Whole genome sequencing reveals the majority of the human genome. WGS has the potential to discover new genes, gene modifiers and the data obtained through genome sequencing would help to address complex inheritance models. This powerful tool makes it possible to discover the genetic cause with a single test, which means that in the future it could become a genetic test of the first choice (Scocchia et al. 2019; Mazzarotto et al. 2020). WGS can detect a broader range of genetic variation than other sequencing approaches, including single-nucleotide variants (SNVs) and insertion or deletions (indels) but also structural variants such as copy-number variants (CNVs) and translocations. Use of the approach is limited due to equipment unavailability, cost and complexity of the genome (Klein and Foroud 2017; Lappalainen et al. 2019; Ho et al. 2020) as well as current bioinformatics limitations to interpret noncoding genomic variation (Zhu et al. 2017; Oakeson et al. 2017; Lionel et al. 2018).

WES and WGS have great potential in the diagnosis of rare diseases, enabling the analysis of many genes in one test, and in the same time resulting in incidental findings and variants of unknown significance (VUS). They represent additional challenges for clinicians and patients.

### Incidental findings

Incidental findings may indicate that a patient is at risk for a disease that is currently not suspected or present and is not related to the patient’s current phenotype. These findings are related to conditions, genes or variants in which some measures could be taken to prevent, postpone or alleviate the disease (Delanne et al. 2019). When data from the entire exome or genome is available, there is always a possibility of an incidental discovery of a pathogenic variant unrelated to the patient phenotype. The American College of Medical Genetics and Genomics (ACMG) has published a list of 59 genes and recommended reporting mutations in these genes back to referring clinicians, regardless of the primary patients’ symptoms (Kalia et al. 2017). The European and Canadian guidelines have taken a more conservative approach and currently recommend limited use of WES and where possible targeted sequencing panel, to reduce the possibility of incidental findings (Claustres et al. 2014; Boycott et al. 2015).

Reporting of incidental findings provides an opportunity for medical intervention, which brings health benefit by allowing early diagnosis, improved disease prediction and prevention. However, there are important concerns due to incomplete disease penetrance and unpredictable disease outcomes, reporting of variants of unknown significance, which could be entirely benign in the disease gene, due to reporting adult-onset disease findings in children, and due to possible negative psychological impact of incidental findings on patients (Hehir-Kwa et al. 2015; Hofmann 2016). In large scale studies, incidental findings were reported with a frequency of up to 6%, which can pose a vast financial burden on a healthcare system (Yang et al. 2013; Burke et al. 2013; Lee et al. 2014; Taylor et al. 2015; Yavarna et al. 2015; Retterer et al. 2016; Seaby et al. 2016; Monies et al. 2019; Al-Dewik et al. 2019). If a laboratory chooses to offer to report incidental findings, a separate consent process is naturally needed (Van El et al. 2013).

### Variants of unknown significance

Given the large amount of data generated by NGS, determining the clinical significance of all variants is challenging. Many of the identified variants can be classified by ACMG standards and guidelines to pathogenic, likely pathogenic, likely benign or benign and a large part of the variants, however, remain in the category of variants of uncertain significance (VUS) (Richards et al. 2015). The potential role of variants in the patient’s diagnosis can be assessed based on their possible presence in databases like gnomAD, ClinVar and HGMD, the characteristics of the variant, comparison of phenotypic traits with those known to be associated with gene variants and search for the variant in healthy and affected family members. The variants of uncertain significance (VUS) known as class 3 according to ACMG classifications are unable to be further classified as either likely benign or likely pathogenic, despite the increasing knowledge and the emergence of international databases. Additionally, the laboratories may opt for VUS subcategories to determine whether or not a VUS may be associated with a patients’ phenotype (Richards et al. 2015; Vears et al. 2017). However, there are web-based tools that through international collaboration, facilitate the exchange of information on VUSs, thereby accelerating and enabling further evidence of pathogenicity for these variants, in addition to offering improvements in the detection of the a disease gene (Australian Genomics Health Aliiance (AGHA) Patient Archive n.d.; DECIPHER n.d.; GeneMatcher n.d.; Monarch Initiative n.d.; MyGene2 n.d.). Variable practices among diagnostics laboratories exist regarding VUS reporting since there are no specific recommendations whether VUS should be reported or not (Rehm et al. 2013; Matthijs et al. 2016; Vears et al. 2017). With time the status of variants classified as VUS is likely to change. Reinterpretation of VUS 12 months later from initial analysis increases diagnostic efficacy. For diagnostic laboratories, it is considered good practice to reissue a medical report if VUS is being reclassified; however, based on a few guidelines, it is not mandatory for laboratories to routinely reanalyse sequence data (Richards et al. 2015; Vears et al. 2017, 2018).

The prevalence of VUS varies significantly in studies. The rate depends on the cohort of patients, the diagnostic method and heterogeneity of rare diseases investigated (Trujillano et al. 2017; Walsh et al. 2020; Mighton et al. 2020).

The unclear outcome of the genetic test creates concern for the VUS carriers and their families and difficulties for clinicians responsible for patient management. The meaning of the term VUS may be challenging to understand for patients and nongenetic clinicians. There is concern that more clinical relevance is given to VUS than is warranted (Vears et al. 2017).

## Implementation of NGS in healthcare system: case of Slovenia

Like other EU countries, especially small ones, Slovenia was heavily dependent on cross-border testing, covering only 62 genes (The European Union Committee of Experts on Rare Diseases n.d.) up to 2013. In this year, a strategic decision to introduce NGS in the healthcare system was made to increase accessibility and speed of diagnosis of genetic disorders and to reduce the dependence on cross-border collaboration. NGS was introduced into clinical practise as part of a routine diagnostic process at the Clinical Institute of Genomics Medicine (CIGM), University Medical Centre Ljubljana which is the national tertiary genetic centre and is currently used as the primary diagnostic tool for more than 51% cases of rare diseases. To assure responsible and efficient use of the new technology and to achieve economic sustainability, NGS testing at CIGM is offered via clinical genetic service; patients referred from various medical specialties are evaluated by a clinical geneticist who checks for appropriateness of the referral (diagnostic hypothesis, probability of genetic aetiology, clinical utility), performs phenotyping of the patient, communicates with the NGS diagnostic unit (Centre for Mendelian Genomics at CIMG) in terms of interpretation of the result and provides pretest and posttest genetic counselling. Clinical and whole exome sequencing is used for all the patients, and different approaches to interpretation are used (bioinformatic panels, phenotype driven gene panels, open exome, trio design) relevant to the clinical context (Ales et al. 2016). For patients with no specific diagnosis, including syndromic cases additionally trained medical doctors and clinical geneticists (in NGS interpretation) are directly involved in the diagnostic in close communication with referring clinical geneticist to bring medical expertise in the diagnostic process. Consequently, the need for multidisciplinary diagnostic meetings involving many specialists is significantly reduced and the time to final diagnosis shortened.

In 2019, whole genome sequencing was introduced in the routine clinical setting for selected cases. With systematic use of WES, we achieved an overall diagnostic yield of 42.2% (Bergant et al. 2018) and nearly 30% in the group of clinically undiagnosed patients with rare diseases. Pathogenic/likely pathogenic variants have been identified in more than 1500 different genes. Up to 40% of pathogenic variants are novel and have not yet been reported in the literature or public databases. The rate of VUS was estimated at 9.3% (Bergant et al. 2018). Pathologic and normal genetic variations are regularly entered in the Slovenian genomic database and submitted to ClinVar.

With the systematic use of NGS approach in the national health system, we provide patients in Slovenia equal access to the comprehensive diagnostic services, and we significantly reduced dependence of national health system on cross-border collaboration. Moreover, during the provision of diagnostic testing for rare diseases, we participated in the identification of several novel genes for human disorders (Writzl et al. 2017; Maver et al. 2017, 2019, 2019; Zaman et al. 2018; Loges et al. 2018; Tolchin et al. 2020).

## Discussion

For single gene disorders with distinguishable phenotype, a traditional genetic test like Sanger sequencing provides high accuracy and achieves the diagnostic goal (Briggs et al. 2001). However, most genetic diseases are not caused by a single gene mutation, nor do they have a recognizable clinical presentation; therefore NGS is quickly replacing traditional methods in the diagnostics of rare genetic diseases.

Diagnostic yield of WES for various heterogeneous diseases is reaching up to 40% in large scale population studies, whereas in consanguineous population the diagnostic yield is up to 80% (Yang et al. 2013, 2014; Lee et al. 2014; Yavarna et al. 2015; Retterer et al. 2016). Limited WGS studies report 40–70% diagnostic yield across various groups of genetic diseases within small cohorts (Mattick et al. 2018; Scocchia et al. 2019).

Comparative study between Sanger sequencing and NGS approaches for various etiologically heterogeneous disorders revealed higher diagnostic yield obtained by NGS (Neveling et al. 2013). Among groups of patients with an unexplained intellectual disability or developmental delay, the yield using a traditional test such as classical karyotyping did not exceed 10%, and an additional 15–20% improvement in diagnostic yield was provided by microarray comparative genomic hybridization (aCGH), which detects genomic copy number variants (Lee et al. 2018; Jang et al. 2019), while NGS improved diagnostic efficacy by up to 62% (Yang et al. 2013; Gilissen et al. 2014; Lee et al. 2014; Iglesias et al. 2014; Al-Dewik et al. 2019).

An important advantage of NGS is well presented in a specific group of patients, where traditional tests have not been successful in making the diagnosis in a timely manner, although it could significantly affect further treatment. Clinical utility of NGS is especially relevant for patients in whom timely diagnosis significantly impacts prognosis and treatment including neonates in the intensive care unit, patients with life-limiting diseases, patients with unrecognized ultrarare diseases, patients with atypical disease presentation and age-dependent penetrance (Stark et al. 2016; Fernandez-Marmiesse et al. 2017; Meng et al. 2017; Elliott et al. 2019).

Despite all the improvements brought by novel technologies, some patients still remain without a diagnosis. According to OMIM statistics, around 12,000 genes are not currently associated with any human disease, and around 2000 phenotypes have a possible unknown molecular genetic basis (OMIM - Online Mendelian Inheritance in Man n.d.). Consequently, to further improve diagnostics of genetic diseases, many efforts have been made towards new gene discovery using new sequencing methods, in particular WGS, yet the yield is lower than initially anticipated (Smedley et al. 2016). Also, the number of undiscovered genes does not correlate with the number of predicted phenotypes awaiting underlying genetic cause. Possibly unresolved genetic diseases may not be recognizable by conventional clinical approaches, underscoring the need for deep phenotyping in conjunction with novel diagnostic approaches, including optical mapping, RNA seq and methylation analysis to improve the diagnosis of rare diseases (Chatterjee et al. 2017; Yip et al. 2018; Bamshad et al. 2019; Chaisson et al. 2019).

Before the introduction of NGS based approaches in clinical practice, the diagnostic capacity varied significantly across European countries. Namely, even in large developed countries, only genetic tests for up to 50% of known genes were available (The European Union Committee of Experts on Rare Diseases n.d.). In order to provide better patient care, cross-border collaboration was necessary. However, due to the limited public health resources in several EU countries, usually, only part of the patients with well-defined phenotypes was typically offered genetic testing abroad. A review of the Orphanet database reveals that at least 336 registered laboratories in Europe are currently providing NGS sequencing (Orphanet n.d.). Numerous countries worldwide are thus enabling cost-effective genetic testing at a national level. Despite the widespread application of NGS, there are still differences in the national/regional genetic testing offered by European countries, and cross-border genetic testing remain necessary to prevent inequalities in patient care (Peterlin et al. 2020). A recent study of cross-border testing reveals unequal access to genetic testing among EU citizens, as some European countries still limit genetic testing at local, national and cross-border level, leaving patients without a genetic diagnosis (Commission Expert Group on Rare Diseases Recommendation 2015; Pohjola et al. 2016).

American College of Medical Genetics and Genomics and Eurogentest/European Society of Human Genetics and Canadian College of Medical Geneticists have published guidelines related to the implementation of NGS in clinical settings (Richards et al. 2015; Matthijs et al. 2016; Hume et al. 2019). Due to the fast development of NGS, recommendations on clinical implementation of NGS are still evolving and at present are characterized by a lack of standardization. In 2014, an evaluation of the practices of 30 international groups using genome sequencing in diagnostic and clinical settings revealed that especially clinical interpretation and outcome reporting lacked the best clinical practices and needed further improvement (Brownstein et al. 2014). Achieving the goals requires the collaboration and education of those involved in the clinical and diagnostic care of patients.

In order to ensure best practice for patients with genetic diseases, it is necessary to establish optimal clinical and analytical pathways at the national level within public healthcare. Clinical pathways should be based on latest guidelines, but a search of the existing literature reveals difficulties in retrieving best clinical practice guidelines for patients with genetic diseases, which are reflected in several recommendations available from various sources (Hilton Boon et al. 2014; Pavan et al. 2017).

NGS is widely accepted method, and some laboratories have adopted NGS as the gold standard for the diagnosis of rare disease; however, current recommendations still require confirmation of a critical NGS finding with Sanger sequencing (Lee et al. 2019; Hartman et al. 2019). Different approaches are performed to identify the cause of genetic diseases. The current recommendations for NGS approaches are based on expert opinion, not evidence. Target panel analysis is usually applied for different heterogeneous groups of genetic disease, and the number of the analysed genes varies across laboratories. WES is commonly exploited in cases with a negative result of targeted sequencing and in cases where a novel phenotype-gene association is expected. WGS is not yet widely used and is generally reserved for selected cases only.

Genetic diseases occur in all fields of medicine. Education is therefore essential for the development and maintenance of genetic competencies, especially as the knowledge of genetics and genomics is rapidly evolving. An important future element is that clinical geneticists play a key role in the education of nongenetic professions; indeed, in the world of science, the art of teaching is to help the others understand (McClaren et al. 2020).

## Conclusion

NGS diagnostic approaches have significantly improved diagnosis of rare diseases and are therefore becoming a first-tier genetic testing tool for several groups of diseases. NGS can significantly improve the access of patients with rare diseases to diagnosis and reduce the dependence of national health systems for cross-border collaboration. Standardization of clinical pathways and technical standards will along with novel NGS applications contribute to further improvement in diagnostics of rare diseases.
